# The evolution of treatment for early breast cancer: what’s next?

**DOI:** 10.3332/ecancer.2009.148

**Published:** 2009-06-09

**Authors:** T Howell

**Affiliations:** Cancer Research Campaign Department of Medical Oncology, University of Manchester, Christie Hospital and Holt Radium Institute, Wilmslow Road, Manchester, M20 4BX, UK

Across Europe, the outlook for women diagnosed with early breast cancer is likely to be far better now than at the start of my career. Effective screening programmes mean women are being diagnosed earlier, and greatly improved surgical techniques have also made a significant impact. Post-surgery, the evolution of adjuvant therapy has meant that we now have an armoury of effective treatments to stop breast cancer returning, although debate continues on how these treatments should be used for maximum benefit.

The greatest concern for doctors, and for women who are being treated for early breast cancer, is that it may return, which ultimately means their disease is incurable. Although all patients are at risk of recurrence, the greatest risk is around 18 months after surgery and a level of risk remains for many years, particularly in hormone receptor positive early disease [1]. Combating recurrence is therefore one of the most important aspects of any treatment strategy and where the focus of clinical investigation has been for many years.

Looking back at clinical trials shows how far the oncology world has come. The effect of oestrogen on breast cancer risk was first shown over 100 years ago when researchers found that removing the ovaries of women with breast cancer improved their chances of survival. Following this, the first anti-oestrogens were developed and it is now over 30 years since tamoxifen was found to delay breast cancer recurrence. In 1983, the breakthrough NATO trial confirmed the place of tamoxifen as a gold-standard treatment for post-menopausal women with early breast cancer, by reducing the risk of recurrence by around 50% [2].

Following tamoxifen, aminoglutethimide was investigated as an anti-aromatase adjuvant treatment for early breast cancer, laying the first steps for aromatase inhibitors (AIs) [3] AIs are a revolutionary class of drugs that work by interrupting the production of oestrogen that can promote tumour growth in post-menopausal women. Anastrozole was the first of the so-called new-generation aromatase inhibitors, introduced in 1995, followed by letrozole and exemestane in 1996 and 1999 respectively, all of which now rival the efficacy of tamoxifen and have fewer serious side effects.

Data from the ATAC (Arimidex, Tamoxifen, Alone, or in Combination) trial, one of the world’s largest and longest-running breast cancer trials, comparing five years of upfront anastrozole with tamoxifen, has further confirmed the place of anastrozole as a new gold-standard treatment for early breast cancer. Over a median follow-up of 100 months (>8 years), anastrozole significantly reduces the risk of recurrence compared with tamoxifen by 24% (p<0.0001) and improves disease-free survival by 15% [4]. One of the most important findings, however, was the significant ‘carry-over’ effect of the drug, that is anastrozole continues to benefit women even four years after treatment ends. It is not truly understood why the positive effect of the drug persists and continues to increase over time, compared with tamoxifen, although what we may be seeing is an effect against the formation of micro-metastases during treatment. To date, anastrozole is the only AI, which has demonstrated this carry-over benefit [4].

Other early breast cancer trials that have followed thousands of women over many years have also confirmed the benefits of AI treatment, indicating that five years of treatment with tamoxifen alone may be sub-optimal for all patients other than those at the lowest risk of recurrence. Recent survival data from BIG 1–98, albeit not statistically significant, suggest longer follow-up and meta-analytical approaches may be required in order to determine whether the efficacy advantages of the aromatase inhibitors over tamoxifen, in terms of recurrence risk reduction, will translate into breast cancer survival benefit [5].

The debate still remains, however, around when it is best to introduce an AI into a treatment regimen, that is upfront as initial adjuvant treatment, following 2–3 years of tamoxifen or to extend adjuvant treatment after five years of tamoxifen is completed. The Intergroup Exemestane Study (IES) reported that switching to exemestane after 2–3 years of treatment with tamoxifen significantly improved survival rates for the AI, although it should be remembered that a switching strategy may put patients at risk by starting with the inferior treatment at the time when risk of recurrence is highest and also expose patients to the risk of side effects from two different agents [6]. The BIG 1–98 study suggested that upfront letrozole was superior to tamoxifen followed by letrozole [5].

To add to the evidence base, a number of investigations are underway to ascertain the optimal treatment period for adjuvant therapy. There is a clear rationale for continuing to treat with hormonal therapy, given that breast cancer recurrence remains a threat more than ten years after surgery. Preliminary results from the Adjuvant Tamoxifen Longer Against Shorter (ATLAS) study investigated the benefits of using tamoxifen beyond five years, although the accumulation of serious side effects during long-term tamoxifen use has led to questions around the effectiveness of this approach [7].

Ensuring women remain free from breast cancer for many years will continue to be a clinical challenge. In my view, the best chance of protecting against recurrence is to commence treatment with the most effective adjuvant therapy at diagnosis, one that is well tolerated, has a well-understood safety profile, and means that fewer patients have to be told the devastating news that their breast cancer has recurred. Advancements in treatments and ongoing clinical debate will undoubtedly continue, but we can be certain that the evolution of hormonal treatments has secured the place of aromatase inhibitors for many years to come.

## Professor Tony Howell

Tony Howell is a Professor of Medical Oncology in the Cancer Research UK Department of Medical Oncology, University of Manchester, at the Christie Hospital NHS Foundation Trust, Manchester, UK. After training in London and Birmingham and working for the Medical Research Council, he became the Head of the Medical Oncology Breast Service, Christie Hospital. His major interest is in all aspects of breast diseases, having published more than 472 papers in this area. He has received educational and research grants from AstraZeneca.
